# Impact of a standardized emergency department asthma care pathway on health services utilization

**DOI:** 10.1186/s13223-025-00973-4

**Published:** 2025-06-11

**Authors:** Chanel Kwok, Katherine Lajkosz, Carole Madeley, Mona Jabbour, Teresa To, M. Diane Lougheed

**Affiliations:** 1 Department of Medicine, University of Ottawa, Ottawa, Canada; 2 Institute for Clinical Evaluative Sciences, Toronto, Canada; 3 The Lung Association- Ontario, Toronto, Canada; 4 Departments of Pediatrics and Emergency Medicine, University of Ottawa, Ottawa, Canada; 5 Dalla Lana Graduate School of Public Health, University of Toronto, Toronto, Canada; 6 Asthma Research Unit, Kingston Health Sciences Centre, Department of Medicine, Queen’s University, 102 Stuart Street, Kingston, ON K7L 2V6 Canada

**Keywords:** Asthma, Emergency department, Care pathway, Health services use

## Abstract

**Background:**

An evidence-based standardized ED asthma care pathway (EDACP) was developed and implemented in Ontario, Canada.

**Objective:**

To determine the impact of EDACP implementation and access to ED asthma management resources and specialists on return ED visits.

**Methods:**

All 173 Ontario hospitals were surveyed regarding their access to community and ED asthma specialists and ED asthma management resources, including EDACP implementation date and status as of August 2017. Survey data were linked to provincial health administrative data to quantify acute health services utilization. A Poisson regression interrupted time series analysis was conducted.

**Results:**

Of the 123 hospitals responding to the survey, 44 (35.8%) had approved the EDACP. Data were analyzed for the 5 years preceding (30,028 asthma visits) and 17 months following (7,916 asthma visits) implementation, with a 3-month implementation black-out period. After controlling for auto-regressive factors, EDACP implementation was associated with a 2% reduction in the absolute rate of return ED visits within 72 h (*p* = 0.0124), and within 7 days (*p* = 0.0295) at teaching hospitals. The same effect was not seen at community hospitals. Peak expiratory flow testing (available at 77% of sites) and spirometry (available at 45% of sites) were associated with 34% (*p* = 0.0071) and 23% (*p* = 0.028) reductions in the odds of return ED visits within 72 h, respectively.

**Conclusion:**

The positive results from this large-scale effort to implement an evidence-based knowledge translation initiative in diverse settings, suggests there is merit in continuing to invest time and resources to overcome barriers to adoption and implementation of this EDACP.

**Supplementary Information:**

The online version contains supplementary material available at 10.1186/s13223-025-00973-4.

## Background

Asthma is a chronic lung disease associated with symptoms of cough, wheeze and shortness of breath. It affects an estimated 262 million individuals globally and 3.8 million Canadians over the age of 1 [1, 2]. Two in three Canadians with asthma over 12 years of age report poorly controlled asthma [3]. Suboptimal control of asthma contributes to increased patient morbidity and health care costs with 20-year direct costs in Canada projected to be $24.40 billion [4]. During an asthma exacerbation many patients visit an emergency department (ED) for management. Return ED visit rates have been used as a measure of the quality of acute asthma care [5].

Strategies for managing asthma in the ED include advanced medical directives, pre-printed orders, objective measures of airflow obstruction (peak expiratory flow (PEF) or spirometry), written discharge instructions and access to specialists when required. Pre-printed physician orders and access to a pediatrician have been associated with lower ED return visit rates in the pediatric asthma population [6]. 

Despite regularly updated national and international asthma guidelines, there continue to be significant discrepancies between the recommended evidence-based care and the care patients receive [7–11]. These disparities likely contribute to poor asthma control, increased patient morbidity and subsequent health services utilization (HSU). The Ontario Ministry of Health and Long-Term Care funded the development of a standardized ED asthma care pathway (EDACP) as an operational version of the Canadian Asthma Consensus Guidelines and Canadian Association of Emergency Physicians / Canadian Thoracic Society guidelines to improve adherence to evidence-based asthma care in the ED [10, 12–15]. 

A pilot study of the EDACP found intervention sites had significantly higher referrals and retention of asthma teaching done during the ED visit [16]. Analysis of patients managed with the EDACP revealed higher documentation of PEF, use of systemic steroids in the ED and on discharge, and involvement of respiratory therapists in ED care. Implementation and uptake of the pathway was challenging due to the diversity of the 5 EDs in the study group. Despite this, the majority of participating staff favoured province-wide dissemination of the EDACP [17]. 

These positive findings supported endorsement by the Canadian Thoracic Society and funding for a provincial ‘roll-out’ of the EDACP led by The Lung Association-Ontario (now known as the Lung Health Foundation) [18, 19] Following knowledge translation principles, regional multi-disciplinary implementation workshops were held throughout the province between the fall of 2008 until 2011 [12]. Subsequently, The Lung Association-Ontario conducted regular surveys of all Ontario EDs to maintain up to date information of implementation status.

We sought to: (1) determine the impact of EDACP implementation on HSU, including hospital admissions and return ED visits; and (2) assess the impact of access to ED asthma management resources and specialists on return ED visits within 72 h and hospital admissions.

## Methods

### Overall design and setting

We performed a retrospective data linkage study of a cohort of adults who were seen in an ED or urgent care centre in Ontario, Canada for asthma using population-based administrative health databases within ICES (formerly Institute for Clinical Evaluative Sciences). ICES is an independent, non-profit research institute whose legal status under Ontario’s health information privacy law allows it to collect and analyze health care and demographic data, without consent, for health system evaluation and improvement. This study was approved by the institutional review board at the Queen’s University Health Sciences and Affiliated Teaching Hospitals Research Ethics Board (Kingston, Ontario, Canada).

### Data sources

**Fig. 1:**
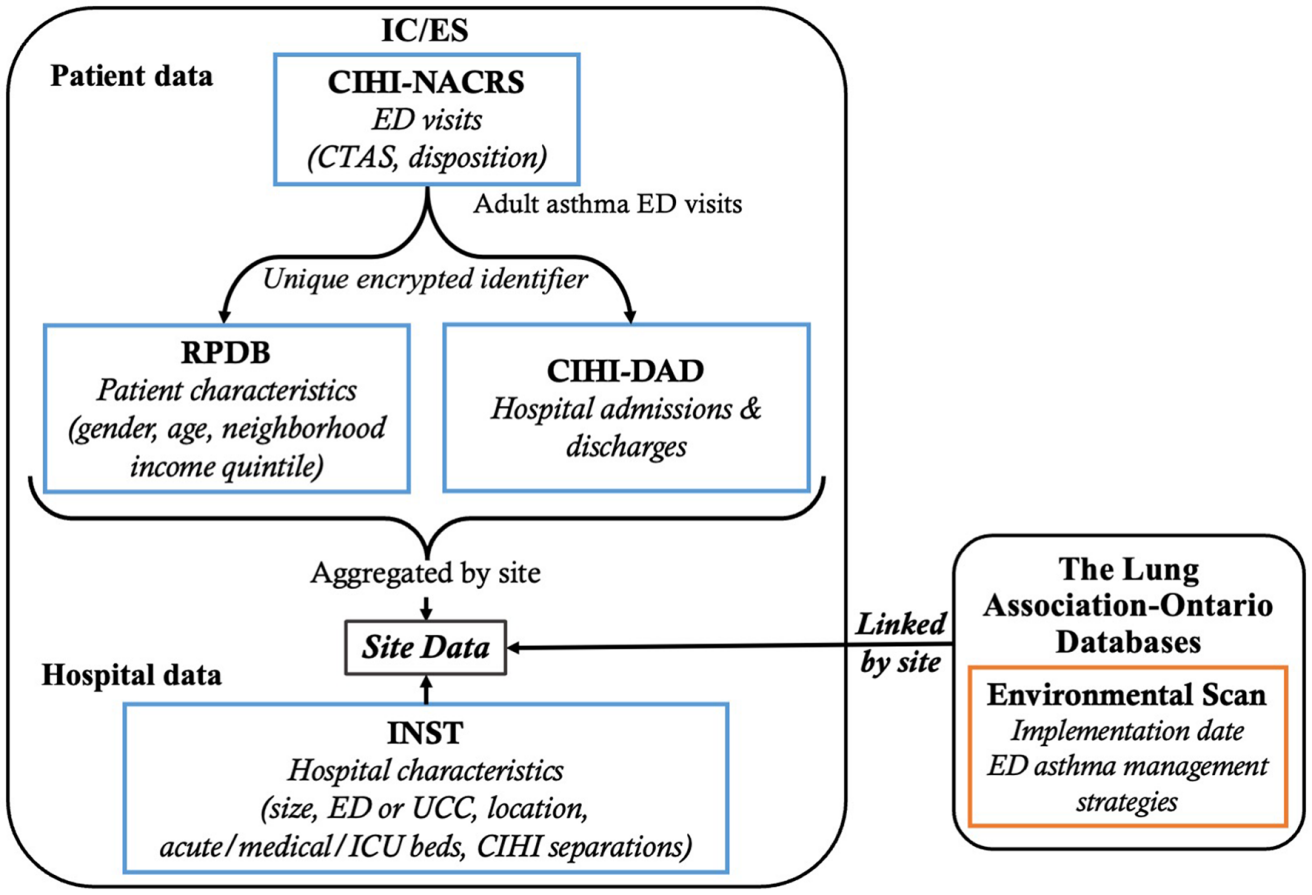
ICES data linkage flow chart. Flowchart showing how ICES datasets and the imported external dataset were linked for site-level analysis

Records of visits to EDs and urgent care centres by adults (20 to 64-years old) for asthma in Ontario were extracted from the Canadian Institute for Health Information (CIHI)-National Ambulatory Care Reporting System within ICES (Fig. 1). Asthma was defined as asthma or status asthmaticus (International Classification of Disease [ICD] 10-CA codes J45 and J46) as the primary disposition diagnosis or as the secondary diagnoses with an additional primary diagnosis of wheeze, dyspnea, cough, or respiratory failure (R06.2, R06.0, R05, J96.0). If a patient was transferred during their visit, the index visit was assigned to the ED from which they were eventually discharged. Return visits were allocated to the index ED site. Patients who died or were missing data were excluded from our analysis.

Unique encrypted identifiers were used to identify multiple visits to EDs by the same individual, extract patient characteristics (sex, age, address) from the Registered Persons Database and records of hospital admissions from the CIHI-Discharge Abstract Database.

A patient’s residential postal code was used to identify the nearest census-based neighbourhood (census dissemination area-average population of 400–700) income quintile. Income quintiles were determined by ranking the average neighbourhood income per single-person equivalent (a household size-adjusted measure of household income) from the 2011 census of each dissemination area within each census metropolitan area or census agglomeration, making them relative to the surrounding neighbourhood, rather than to the whole province. Exacerbation severity was based on Canadian Triage and Acuity Scale assigned at the index visit.

Hospital characteristics were obtained from the institution database. Hospitals were defined as being either teaching (associated with a medical school), community (non-teaching hospitals with at least 45 beds), or small (non-teaching hospitals with less than 45 beds). The ED visit disposition was used to determine propensity to admit. The numerator was the number of ED visits for asthma that resulted in an inpatient admission (ED visit disposition was admission to hospital) divided by the total ED visits for asthma. This was calculated over a one-year lookback period. The overall number of ED visits and hospital admissions was used as a marker of hospital volume. Sites with missing data were excluded from the analysis. All data were aggregated by site for analysis.

The Lung Association-Ontario conducted detailed surveys of all Ontario EDs in 2016 and 2017 to obtain current pathway implementation status, information on access to community and ED asthma specialists and ED asthma management resources. This information was entered into a database and linked to the aggregated site-level data within ICES.

### Statistical analysis

All analyses were carried out using SAS Enterprise guide 6.1 (SAS Institute Inc., Cary, NC) and conducted within ICES.

#### Pathway implementation

For sites with a reported EDACP implementation date, records of ED visits were obtained for the 5 years preceding and 17 months (most recent available) following implementation at each site, with a 3-month blackout period at the time of implementation. Sites without an implementation date or insufficient follow-up data were excluded from the analysis. Given the varying dates of pathway implementation a Poisson regression interrupted time series (ITS) analysis was performed, allowing each site’s pre-implementation period to be its own control [20]. 

#### ED and community asthma resources

For sites that responded to the resource availability survey, records of ED visits were obtained for the fiscal year 2015 (April 1, 2015 to March 30, 2016). Given the nested nature of patients within institutions, hierarchical logistic regression models were used to investigate the association between ED and community asthma resources and: (a) return ED visits within 72 h; (b) subsequent hospital admission.

Patient-level confounders assessed included sex, age, neighborhood income quintile, exacerbation severity, and recent ED visit or hospitalization for asthma within the 12 months preceding the index ED visit. Site-level confounders assessed included hospital type, propensity to admit, proportion of asthma-related ED patients (per 100 ED patients), and volume of all ED visits.

## Results

### Pathway implementation

Forty-four sites reported having implemented the EDACP as of August 2017. Nine sites were excluded from the ITS analysis due to an unknown implementation date or insufficient follow-up data. Table 1 shows the characteristics of the hospitals by implementation status. Over half of the hospitals reporting EDACP implementation were medium-sized community hospitals. Fewer small hospitals (23.8%) and more teaching hospitals (61.1%) reported implementation and therefore the implementation group had significantly more beds (total, medical and critical care).

**Table 1:** Hospital characteristics by implementation status

Characteristic	Non-implementers (*n* = 79)	All implementers (*n* = 44)	Implementers for ITS analysis (*n* = 35)
Hospital type*			
Teaching	7 (8.9%)	11 (25%)	7 (20.0%)
Community	40 (50.6%)	23 (52.3%)	20 (57.1%)
Small	32 (40.5%)	10 (22.7%)	8 (22.9%)
ED	73 (92.4%)	41 (93.2%)	33 (94.3%)
Open 24/7	76 (96.2%)	42 (95.5%)	34 (97.1%)
Has acute care beds	74 (93.7%)	41 (93.2%)	31 (88.6%)
Medical beds, mean (SD)*	41.2 (57.3)	88.9 (108.6)	81.7 (97.4)
ICU beds, mean (SD)*	7.4 (9.6)	20.3 (27.7)	18.6 (25.5)
Total beds, mean (SD)*	93.9 (106.6)	198.1 (243.7)	176.8 (206.5)

Abbreviations: ITS-interrupted time series; ED-emergency department; ICU-intensive care unit; SD-standard deviation

*p < 0.05 for implementers vs. non-implementers

At the 35 implementer hospitals, in the 60 months pre-implementation, there were a total of 30,028 asthma episodes (comprised of 22,198 individuals) and in the 17 months post-implementation, there were a total of 7,916 asthma episodes (comprised of 6,819 individuals).(Supplementary Table 1, Additional File 1).

**Fig. 2:**
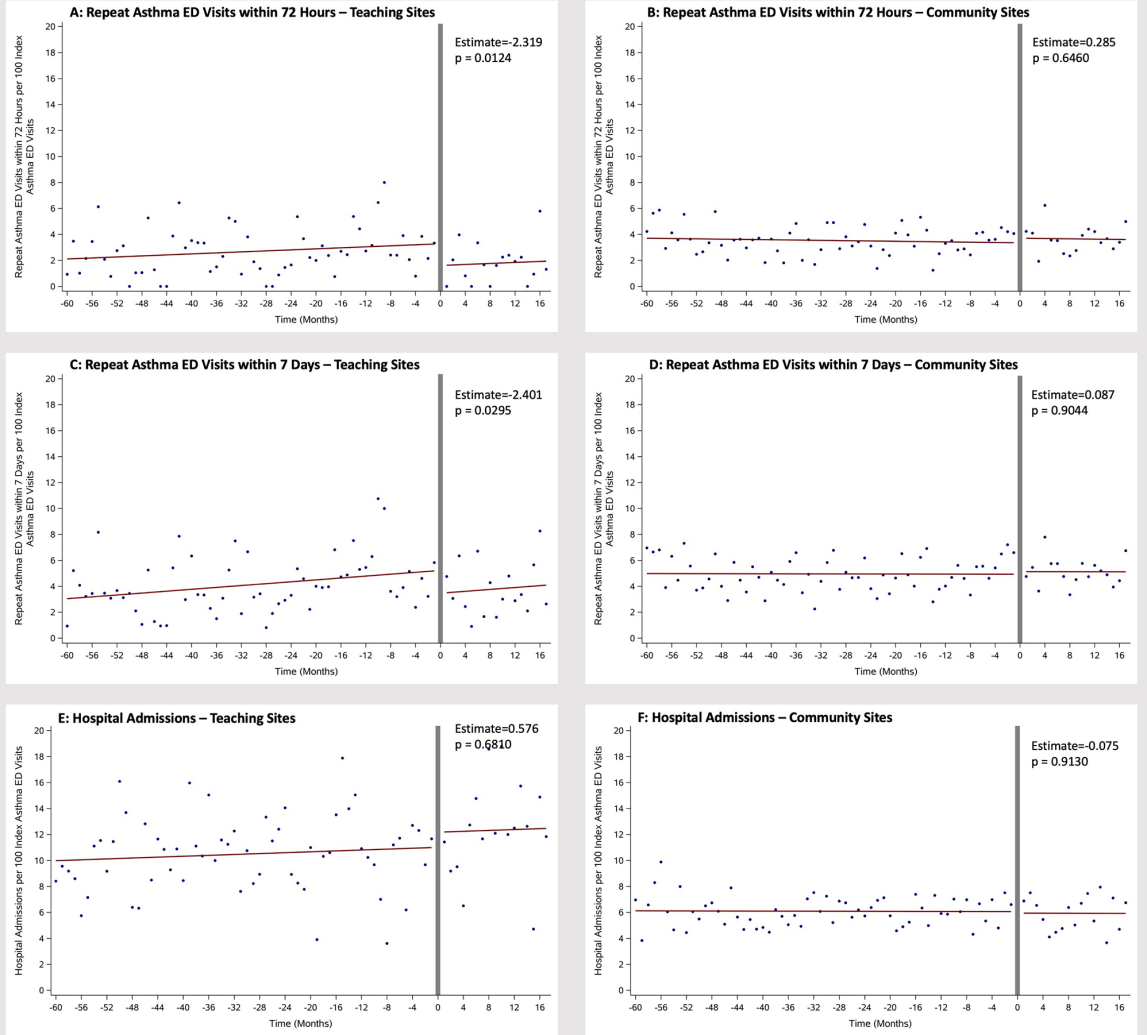
ITS analysis of repeat ED visits and hospital admissions by hospital type for EDACP implementation. Repeat ED visits before and after EDACP implementation with 3 month blackout period (grey line) at teaching hospitals (**A**-within 72 h, **C**-within 7 days) and community hospitals (**B**-within 72 h, **D**-within 7 days). Hospital admissions resulting from index ED visit at teaching hospitals (**E**) and community hospitals (**F**) before and after EDACP implementation with 3 month blackout period (grey line)

ITS analysis for return ED visits and hospital admission by teaching and community (small and medium-sized) hospitals are shown in Fig. 2. After controlling for the baseline trend of increasing repeat visits, at teaching hospitals only, there was a significant decrease in repeat visit rates. For repeat ED visits within 72 h at teaching sites (Fig. 2A), this represented decrease of 2.32% repeat ED visits (*p* = 0.012) following implementation. For repeat ED visits within 7 days at teaching sites (Fig. 2C), this represented a decrease of 2.4% repeat visits (*p* = 0.029) following implementation. These correspond to an absolute reduction of more than 2 per 100 asthma ED visits post-implementation at teaching hospitals within both 72 h and 7 days. No significant differences were seen for repeat ED visits at community hospitals or for hospital admissions for any hospital type.

### ED and community asthma resources

A total of 26,086 patients presented to the 119 (15-teaching, 63-community, 41-small; Table 2) institutions who responded to The Lung Association-Ontario survey. There were no significant difference in hospital characteristics between survey responders and non-responders (Supplementary Table 2, Additional File 1). Patients who died during their index ED visit (*N* = 74) or were missing a triage level (*N* = 56) were excluded from the analysis. Six institutions responded “Unknown” to all ED asthma resource questions. Including the “Unknown” response to the ED asthma resource questions resulted in multicollinearity issues with the models. To circumvent the multicollinearity issues, these six institutions (607 patients) were excluded from the analysis. The model therefore contains a total of 25,349 patients who presented to 113 different institutions that responded to the survey.

**Table 2:** Patient characteristics by hospital type of index ED visit: March 2015 - February 2017

Characteristic	Teaching hospitals (*N* = 6215)	Large community hospitals (*N* = 25035)	Small community hospitals (*N* = 4704)	*p*-value
Female	4031 (64.9%)	15,991 (63.9%)	2988 (63.5%)	0.268
Age (years)				< 0.001
18–34	2479 (39.%)	8964 (35.8%)	1600 (34%)	
35–49	1538 (24.7%)	6254 (25%)	1256 (26.7%)	
50–64	1245 (20%)	5535 (22.1%)	1136 (24.1%)	
≥ 65	953 (15.3%)	4282 (17.1%)	712 (15.1%)	
Neighbourhood income quintile				< 0.001
1	1882 (30.3%)	6313 (25.2%)	994 (21.2%)	
2	1295 (20.8%)	5494 (21.9%)	1012 (21.5%)	
3	1066 (17.2%)	5035 (20.2%)	923 (19.6%)	
4	969 (15.6%)	4691 (18.7%)	922 (19.6%)	
5	939 (15.1%)	3396 (13.6%)	753 (16%)	
Missing	64 (1%)	106 (0.4%)	100 (2.1%)	
CTAS score				< 0.001
1–2 (resuscitation, emergent)	2061 (33.2%)	6819 (27.2%)	354 (7.5%)	
3 (urgent)	3223 (51.9%)	13,296 (53.1%)	1877 (39.9%)	
4–5 (semiurgent, nonurgent)	924 (14.9%)	4904 (19.6%)	2414 (51.3%)	
Unknown	7 (0.1%)	16 (0.1%)	59 (1.3%)	
Disposition				< 0.001
Admitted	568 (9,1%)	1933 (7.7%)	87 (1.8%)	
Died	0	1–5*	1–5*	
Discharged	5480 (88.2%)	22,834 (91.2%)	4583 (97.4%)	
Left before visit completed	115 (1.9%)	216 (0.9%)	26 (0.6%)	
Transferred	52 (0.8%)	47–51*	3–7*	
Admission for asthma within previous 12 months	92 (1.5%)	327 (1.3%)	24 (0.5%)	< 0.001
ED visit for asthma within previous 12 months	348 (5.6%)	1584 (6.3%)	299 (6.4%)	0.093
Return ED visit within 72 h of index visit	105 (1.7%)	602 (2.4%)	148 (3.1%)	< 0.001

Abbreviation: ED-emergency department, CTAS-Canadian Triage Assessment Score

*values suppressed due to small size

Survey responses are displayed in Table 3. There was significant variability in available asthma resources with no single resource being universally available at all survey responder sites. Few sites reported having nearby (< 100 km) access to outpatient specialists (allergists 34.5%, respirologists 31.1%, general internists 20.2%), while an asthma educator was within 100 km of 53 sites (44.5%). Many sites reported availability of PEF meters (77.3%), advanced medical directives for bronchodilators (63.9%), and pre-printed orders (55.5%).

Logistic regression and multi-level logistic regression models for repeat ED visits within 72 h and hospital admission by available ED and community resources are presented in Table 3 (unadjusted models in Supplementary Tables 3 and 4, Additional File 1). The multi-level model grouped patients by institution. Availability of a respirologist for consultation on ED patients was associated with a significantly increased odds of a repeat ED visit within 72 h (OR 1.39, 95% CI:1.08–1.78). Objective measures of airflow obstruction were associated with reduced odds of a repeat ED visit (PEF meter OR 0.69, 95% CI:0.53–0.90; spirometry OR 0.77, 95%CI:0.63–0.95). No significant associations were found in the multi-level logistic regression model for hospital admissions.

**Table 3:** Adjusted ORs* for return ED visits within 72 h and hospital admissions, by asthma management strategies and resources (*N* = 113)^*†*^

Resources and management strategies	Number of sites	Return ED visit within 72 h OR (95% CI)	Hospital admission OR (95% CI)
**Community asthma resources**			
Allergist	78 (65.5%)	0.93 (0.69–1.27)	0.78 (0.54–1.11)
Asthma educator	66 (55.5%)	1.20 (0.96–1.50)	1.07 (0.83–1.38)
Respirologist	82 (68.9%)	0.95 (0.66–1.37)	0.98 (0.65–1.46)
General internist	95 (79.8%)	0.87 (0.58–1.30)	0.54 (0.41–1.02)
**ED asthma specialists**			
Respirology	35 (29.4%)	1.39 (1.08–1.78)	0.82 (0.63–1.07)
General internal medicine	75 (63%)	0.84 (0.61–1.16)	1.09 (0.73–1.63)
Respiratory therapy	75 (63%)	0.92 (0.63–1.32)	1.34 (0.81–2.24)
Anesthesiology	92 (77.3%)	0.88 (0.58–1.33)	0.61 (0.34–1.11)
**ED asthma resources**			
Peak expiratory flow testing	92 (77.3%)	0.66 (0.49–0.89)	1.38 (0.97–1.97)
Spirometry	54 (45.4%)	0.77 (0.62–0.97)	1.22 (0.95–1.55)
Advanced medical directives for bronchodilators	76 (63.9%)	0.93 (0.70–1.24)	0.82 (0.61–1.11)
Advanced medical directives for steroids	27 (22.7%)	1.17 (0.89–1.54)	1.10 (0.84–1.43)
Pre-printed orders	66 (55.5%)	1.01 (0.81–1.26)	1.19 (0.93–1.51)
Written discharge instructions	45 (37.8%)	0.98 (0.76–1.25)	0.82 (0.63–1.05)

* Multi-level model with patients grouped by institution and adjusted for: age, sex, neighbourhood income quintile, triage level, ED visits for asthma in the previous 12 months, hospital admissions for asthma in the previous 12 months, hospital type, ED visit volume, proportion of all ED visits with asthma, propensity to admit

^†^Data not provided by six survey respondents

## Discussion

This large-scale effort to implement an evidence-based knowledge translation initiative in diverse settings resulted in approximately one quarter of all EDs in Canada’s largest province implementing a standardized adult asthma pathway. Implementation was associated with significantly reduced repeat ED visits at teaching hospitals. Outpatient asthma specialists and asthma educators were not available within 100 km at the majority of sites. Access to objective measures of airflow obstruction were not universally available in EDs, but when present, were associated with reduced odds of return ED visits.

To the best of our knowledge, this is the first large scale study assessing an ED care pathway implemented across multiple diverse sites. The initial pilot study evaluating the EDACP showed a trend toward reduced return ED visits at 24 h, 72 h, and 7 days [16]. However, those differences were not statistically significant as the study was not powered for this outcome. Our study represents the next step in evaluating the effect of the EDACP after a larger scale implementation initiative and showed significantly reduced ED return visits at 72 h and 7 days at teaching hospitals following EDACP implementation. Furthermore, this visit rate reduction is almost equal to the previously described baseline repeat visit rate [21]. 

Gaps between guideline-recommended care and clinical practice have previously been identified for treatment of asthma in the ED [9, 22–25]. The EDACP aims to provide an operational version of evidence-based guideline care to improve acute asthma care and prevent relapse following exacerbations. Our study continued to demonstrate that although both guidelines and the EDACP recommend objective measurements of airflow obstruction to assess the severity of an exacerbation and monitor response to treatment [7, 10, 14], PEF was not available at nearly 1 in 4 sites, despite its low cost and portability. Poor subjective assessment of airflow obstruction has previously been associated with increased risk of ED visit, further confirming the need of objective testing [26]. Providing the appropriate tools for accurate assessments is an additional barrier to optimal care that needs to be addressed.

The strengths of this study include linking site-level survey data with administrative data to allow objective evaluation of HSU across multiple sites, including capturing return visits made to different sites from the index ED visit. Using administrative data also allowed us to evaluate EDACP implementation and HSU outcomes at both larger tertiary care centres and small community hospitals, which are less frequently assessed in studies. Interestingly, we did not find a significant difference in repeat ED visits or hospital admissions in small community hospitals following EDACP implementation. Small community hospitals represented 8 of 10 sites in the pilot study [16], but may not have had as much support for pathway uptake following implementation outside of an intervention study, thus limiting the potential to find the same signal seen at teaching hospitals.

The limitations of our study relate to using survey and administrative data. Date of EDACP implementation was based on survey responses. Every effort was made to obtain a response from all 173 EDs and UCCs in Ontario from a knowledgeable responder such as nurses or physicians in ED administrative leadership roles to ensure responses were accurate. Using administrative data, we are unable to determine which patients seen at sites following EDACP implementation were actually managed with the pathway. In the pilot study there was considerable variability of use at intervention sites (6–60%) [16]. However it is possible that having the pathway implemented at a site would lead to increased use of the pathway’s principles even if the pathway was not directly used. This may explain the findings in the intention to treat analysis from the pilot study showing increased use of inhaled bronchodilators and steroids, delivery and documentation of asthma education in the ED and referrals to specialized asthma services on discharge at intervention sites even when not all patients were managed on the EDACP. Given the variability in use of the EDACP, it is possible that further reductions in repeat ED visits may be seen with higher adoption of the pathway.

Asthma exacerbations reflect sub-optimal control of asthma and repeat ED visits may reflect sub-optimal acute asthma care. Although a number of factors have been associated with increased risk of repeat ED visits [27, 28], few studies have been able to demonstrate interventions that reduce this risk [6, 29]. Our study demonstrated that EDACP implementation significantly reduced repeat visits. This can help to lower patient morbidity and health system costs for patients with asthma and provides support for further initiatives to support implementation and use of the EDACP. Our findings are consistent with similar research in the pediatric population, where implementation of asthma clinical pathways in the ED have been associated with trends towards reduced return visits to the ED [30–33]. 

Our study also identified important gaps in ED asthma care which can be targeted for improvement, including increased access to objective measures of airflow obstruction and increased outpatient access to asthma education and asthma specialists. Given the expansion of virtual care that has occurred during the COVID-19 pandemic [34], this presents an opportunity for specialists to reach patients who typically have less access to specialist services due to geographic location. Our study also found that patients presenting to sites which had access to Respirologists in the ED had a higher odds of repeat ED visits. Although we were not able to assess the characteristics of these patients in any post-hoc analysis, it is possible that those with more severe asthma, who are at higher risk for relapse, tend to present to sites with speciality services.

## Conclusion

Reducing repeat ED visits for asthma is important to reduce patient morbidity and health system costs. Our study demonstrates how a guideline-based EDACP which has been implemented on a large-scale across many diverse settings, can improve asthma ED care and reduce HSU. These findings justify future investments to overcome barriers to uptake and use of this EDACP.

## Electronic supplementary material

Below is the link to the electronic supplementary material.

Supplementary Material 1

## Data Availability

The dataset from this study is held securely in coded form at ICES. While legal data sharing agreements between ICES and data providers (e.g., healthcare organizations and government) prohibit ICES from making the dataset publicly available, access may be granted to those who meet pre-specified criteria for confidential access, available at https://www.ices.on.ca/DAS (email: das@ices.on.ca). The full dataset creation plan and underlying analytic code are available from the authors upon request, understanding that the computer programs may rely upon coding templates or macros that are unique to ICES and are therefore either inaccessible or may require modification.

## Abbreviations

CIHI: Canadian Institute for Health Information
ED: Emergency department
EDACP: Emergency department asthma care pathway
HSU: Health services utilization
ICES: Institute for Clinical Evaluative Sciences
ITS: interrupted time series
PEF: peak expiratory flow
