# Supplementary material for: Impact of a standardized emergency department asthma care pathway on health services utilization
Source: Allergy Asthma Clin Immunol. 2025 Jun 11;21:27. doi: 10.1186/s13223-025-00973-4 (PMC12153106; doi:10.1186/s13223-025-00973-4)
Supplement: Supplementary file 1 — Supplementary Material 1 [file 13223_2025_973_MOESM1_ESM.docx]

Impact of a standardized Emergency Department asthma care pathway on health services utilization

Authors: Chanel Kwok, Katherine Lajkosz, Carole Madeley, Mona Jabbour, Teresa To, M. Diane Lougheed

Additional file 1

**Supplementary Table 1- Patient characteristics by implementation period (pre-implementation 5 years; post-implementation 17 months)**

| **Characteristic** | **Pre-implementation, 5 years (N=30,028)** | **Post-implementation, 17 months (N=7,916)** | **p-value** |
| --- | --- | --- | --- |
| Age (Mean ± SD) | 41.2 ± 17 | 42.2 ± 17.6 | <.001 |
| Female | 18,693 (62.3%) | 4,966 (62.7%) | 0.431 |
| ED Visit for Asthma in Prior Year | 6,876 (22.9%) | 1,744 (22.0%) | 0.101 |
| Neighbourhood income quintile |  |  |  |
| 1 (lowest) | 9,087 (30.3%) | 2,384 (30.1%) | 0.625 |
| 2 | 6,739 (22.4%) | 1,736 (21.9%) |  |
| 3 | 5,596 (18.6%) | 1,447 (18.3%) |  |
| 4 | 4,554 (15.2%) | 1,239 (15.7%) |  |
| 5 (highest) | 3,865 (12.9%) | 1,060 (13.4%) |  |
| Regional health authority |  |  |  |
| 1 | 2,170 (7.2%) | 530 (6.7%) | <.001 |
| 2 | 1,822 (6.1%) | 488 (6.2%) |  |
| 3 | 5,144 (17.1%) | 1,395 (17.6%) |  |
| 4 | 2,149 (7.2%) | 630 (8.0%) |  |
| 5 | 174 (0.6%) | 58 (0.7%) |  |
| 6 | 264 (0.9%) | 69 (0.9%) |  |
| 7 | 3,022 (10.1%) | 869 (11.0%) |  |
| 8 | 461 (1.5%) | 124 (1.6%) |  |
| 9 | 5,794 (19.3%) | 1,519 (19.2%) |  |
| 10 | 1,630 (5.4%) | 411 (5.2%) |  |
| 11 | 4,183 (13.9%) | 1,001 (12.6%) |  |
| 12 | 513 (1.7%) | 204 (2.6%) |  |
| 13 | 2,507 (8.3%) | 546 (6.9%) |  |
| 14 | 168 (0.6%) | 69 (0.9%) |  |

**Supplementary Table 2-Hospital characteristics by survey completion**

| **Characteristics, n(%)** | **Completed survey (N=119)** | **Did not complete survey (N=54)** | **p-value** |
| --- | --- | --- | --- |
| Hospital type |  |  | 0.989 |
| Small community | 41 (34.5%) | 18 (33.3%) |  |
| Large community | 63 (52.9%) | 29 (53.7%) |  |
| Teaching | 15 (12.6%) | 7 (13.0%) |  |
| Urgent Care Centre | 9 (7.6%) | ≤ 5 | 0.705 |
| Open 24/7 | 114 (95.8%) | 51 (94.4%) | 0.694 |
| Has Acute Beds | 111 (93.3%) | 52 (96.3%) | 0.43 |
| Medical Beds, mean±SD | 58.06 ± 84.7 | 39.7 ± 66.3 | 0.17 |
| ICU Beds, mean±SD | 11.7 ± 19 | 8.7 ± 16.5 | 0.324 |
| Total Beds, mean±SD | 129.3 ± 177.4 | 95.7 ± 136.8 | 0.23 |
| ED Visit Volume, mean±SD | 35,715 ± 27,542.1 | 28,785.8 ± 28,348.3 | 0.131 |
| Hospital Admission Volume, mean±SD | 7,834.5 ± 10,334.3 | 5,800.1 ± 9,125.9 | 0.23 |

**Supplementary Table 3-Unadjusted and adjusted ORs (95% CI) for return ED visits within 72 hours**

|  | **Logistic Regression Model** | | **Multi-Level Logistic Regression Model** | |
| --- | --- | --- | --- | --- |
|  | **Unadjusted** | **Adjusted*** | **Unadjusted** | **Adjusted*** |
| **Community Asthma Resources*** |  |  |  |  |
| Allergist | 0.93 (0.76-1.13) | 0.93 (0.70-1.24) | 0.91 (0.73-1.14) | 0.93 (0.69-1.27) |
| Asthma Educator | 1.00 (0.85-1.19) | 1.19 (0.96-1.46) | 0.99 (0.81-1.20) | 1.20 (0.96-1.50) |
| General Internist | 0.85 (0.64-1.13) | 0.89 (0.62-1.29) | 0.80 (0.58-1.10) | 0.87 (0.58-1.30) |
| Respirologist | 0.92 (0.74-1.14) | 0.95 (0.68-1.32) | 0.90 (0.71-1.14) | 0.95 (0.66-1.37) |
| **ED Asthma Services**** |  |  |  |  |
| Anesthesiology | 0.71 (0.53-0.94) | 0.88 (0.60-1.30) | 0.70 (0.52-0.95) | 0.88 (0.58-1.33) |
| General Internal Medicine | 0.77 (0.62-0.94) | 0.83 (0.62-1.12) | 0.75 (0.60-0.94) | 0.84 (0.61-1.16) |
| Respirology | 1.03 (0.88-1.22) | 1.38 (1.11-1.73) | 1.01 (0.84-1.22) | 1.39 (1.08-1.78) |
| Respiratory Therapy | 0.71 (0.58-0.88) | 0.91 (0.64-1.28) | 0.71 (0.56-0.89) | 0.92 (0.63-1.32) |
| **ED Asthma Resources** |  |  |  |  |
| Peak Flows | 0.75 (0.60-0.93) | 0.70 (0.55-0.90) | 0.73 (0.57-0.93) | 0.69 (0.53-0.90) |
| Spirometry | 0.90 (0.76-1.05) | 0.78 (0.64-0.94) | 0.87 (0.72-1.05) | 0.77 (0.63-0.95) |
| Advanced medical directives for bronchodilators | 1.11 (0.92-1.34) | 0.97 (0.76-1.24) | 1.06 (0.85-1.31) | 0.94 (0.72-1.23) |
| Advanced medical directives for steroids | 1.10 (0.93-1.30) | 1.21 (0.96-1.52) | 1.05 (0.86-1.28) | 1.21 (0.93-1.56) |
| Pre-printed orders | 1.10 (0.93-1.30) | 1.04 (0.86-1.26) | 1.06 (0.88-1.29) | 1.02 (0.83-1.26) |
| Written discharge instructions | 0.96 (0.82-1.13) | 0.97 (0.81-1.16) | 0.95 (0.79-1.15) | 0.97 (0.79-1.19) |

***Adjusted models: Adjusted for the following patient characteristics: age, sex, triage level, ED visit for asthma in the previous 12 months, hospital admission for asthma in the previous 12 months, ED visit volume, proportion of all ED visits with asthma. The multi-level model groups patients by institution*

**Supplementary Table 4- Unadjusted and adjusted ORs for return hospital admissions**

|  | **Logistic Regression Model** | | **Multi-Level Logistic Regression Model** | |
| --- | --- | --- | --- | --- |
|  | **Unadjusted** | **Adjusted*** | **Unadjusted** | **Adjusted*** |
| **Community Asthma Resources*** |  |  |  |  |
| Allergist | 1.49 (1.30-1.72) | 0.76 (0.59-0.96) | 1.52 (1.00-2.32) | 0.78 (0.54-1.11) |
| Asthma Educator | 1.34 (1.20-1.49) | 1.13 (0.98-1.31) | 1.36 (0.93-2.00) | 1.07 (0.83-1.38) |
| General Internist | 1.26 (1.03-1.53) | 0.77 (0.57-1.03) | 1.11 (0.63-1.96) | 0.64 (0.41-1.02) |
| Respirologist | 1.49 (1.28-1.73) | 0.93 (0.70-1.22) | 1.53 (0.98-2.38) | 0.98 (0.65-1.46) |
| **ED Asthma Services**** |  |  |  |  |
| Anesthesiology | 3.74 (2.63-5.33) | 0.63 (0.37-1.06) | 2.34 (1.35-4.09) | 0.61 (0.34-1.11) |
| General Internal Medicine | 2.48 (2.06-2.98) | 1.12 (0.87-1.44) | 2.38 (1.57-3.61) | 1.09 (0.73-1.63) |
| Respirology | 1.90 (1.72-2.10) | 0.90 (0.78-1.04) | 2.37 (1.67-3.36) | 0.82 (0.63-1.07) |
| Respiratory Therapy | 4.82 (3.69-6.29) | 1.36 (0.89-2.08) | 3.40 (2.26-5.14) | 1.34 (0.81-2.24) |
| **ED Asthma Resources** |  |  |  |  |
| Peak Flows | 2.23 (1.84-2.69) | 1.19 (0.95-1.49) | 2.17 (1.32-3.58) | 1.19 (0.85-1.67) |
| Spirometry | 1.15 (1.04-1.27) | 1.28 (1.13-1.46) | 1.50 (1.03-2.17) | 1.22 (0.97-1.54) |
| Advanced medical directives for bronchodilators | 1.23 (1.10-1.39) | 0.92 (0.78-1.08) | 1.24 (0.82-1.87) | 0.86 (0.65-1.14) |
| Advanced medical directives for steroids | 1.17 (1.06-1.30) | 1.05 (0.92-1.20) | 1.22 (0.80-1.88) | 1.08 (0.84-1.40) |
| Pre-printed orders | 1.28 (1.16-1.42) | 1.21 (1.05-1.39) | 1.41 (0.96-2.06) | 1.18 (0.94-1.49) |
| Written discharge instructions | 1.23 (1.12-1.36) | 0.89 (0.80-1.01) | 1.34 (0.92-1.96) | 0.86 (0.69-1.06) |

**Adjusted models: Adjusted for the following patient characteristics: age, sex, triage level, ED visit for asthma in the previous 12 months, hospital admission for asthma in the previous 12 months, ED visit volume, proportion of all ED visits with asthma. The multi-level model groups patients by institution*
